# Mutational Analysis of Oncogenic AKT1 Gene Associated with Breast Cancer Risk in the High Altitude Ecuadorian Mestizo Population

**DOI:** 10.1155/2018/7463832

**Published:** 2018-07-03

**Authors:** Andrés López-Cortés, Paola E. Leone, Byron Freire-Paspuel, Nathaly Arcos-Villacís, Patricia Guevara-Ramírez, Felipe Rosales, César Paz-y-Miño

**Affiliations:** ^1^Centro de Investigación Genética y Genómica, Facultad de Ciencias de la Salud Eugenio Espejo, Universidad Tecnológica Equinoccial, Avenue Mariscal Sucre, 170129 Quito, Ecuador; ^2^Escuela de Medicina, Facultad de Ciencias de la Salud, Universidad de las Américas, Avenue de los Granados, 170125 Quito, Ecuador; ^3^Departamento de Ciencias de la Vida, Universidad de las Fuerzas Armadas (ESPE), Avenue General Rumiñahui, 1715231B Sangolquí, Ecuador; ^4^The James Black Centre, Cardiovascular Division, King's College London, BHF Centre of Excellence, 125 Coldharbour Lane, London SE5 9NU, UK; ^5^Departamento de Patología, Hospital Oncológico Solon Espinosa Ayala, Avenue Eloy Alfaro, 170138 Quito, Ecuador

## Abstract

Breast cancer is the leading cause of cancer-related death among women worldwide. AKT1 encodes the kinase B alpha protein. The rs121434592, rs12881616, rs11555432, rs11555431, rs2494732, and rs3803304 single nucleotide polymorphisms have been identified in the AKT1 kinase gene. Activated AKT1 phosphorylates downstream substrates regulating cell growth, metabolism, apoptosis, angiogenesis, and drug responses. It is essential to know how breast cancer risk is associated with histopathological and immunohistochemical characteristics and genotype polymorphisms in a high altitude Ecuadorian mestizo population. This is a retrospective case-control study. DNA was extracted from 185 healthy and 91 affected women who live 2,800 meters above sea level. Genotypes were determined by genomic sequencing. We found a possible association between the noncoding intronic variant rs3803304 and breast cancer risk development: GG (odds ratio [OR] = 5.2; 95% confidence interval [CI] = 1.3-20.9;* P* ≤ 0.05;* Q* > 0.05). Regarding pathologic characteristics, we found significant risk between estrogen receptor, progesterone receptor, and HER2 status and molecular subtypes (*P* ≤ 0.001;* Q* ≤ 0.05). On the other hand, we did not find risk between variants and histopathological characteristics. Despite the small sample size, we found that the intronic variant, AKT1 rs3803304, may act as a predictive biomarker in the risk of developing breast cancer in the high altitude Ecuadorian mestizo population.

## 1. Introduction

Breast cancer (BC) in women involves the progressive accumulation of genetic, hereditary, hormonal, and environmental factors representing a significant health problem worldwide [1]. BC is the leading cause of cancer-related death among women (521,541 cases) and the most commonly diagnosed cancer (1,679,076 cases) [2]. The areas with a higher incidence of BC per each 100,000 inhabitants are Western Europe (89.9), Oceania (85.5), and Northern Europe (76.7), while South America has a lower incidence (44.3) [3]. In Ecuador, the incidence rate of BC has reached up to 32.7 in 2012 [4].

The molecular subtypification of the progesterone receptor (PR), estrogen receptor (ER), and HER2 status coupled with the histopathological classification (noninvasive or “*in situ*” carcinoma and invasive or infiltrating carcinoma) generates five different subtypes: luminal A (ER+ and/or PR+, HER2-, or low Ki67), luminal B (ER+ and/or PR+, HER2+, or HER2- with high Ki67), HER2-enriched (ER-, PR-, or HER2+), basal-like (ER-, PR-, HER2-, cytokeratin 5/6+, and/or HER1+), and normal-like (ER+ and/or PR+, HER2-, low Ki67, prognosis slightly worse than luminal A) [5–7].

Approximately 10% of BC cases correspond to hereditary factors with germline mutations in BRCA1, BRCA2, TP53, E-Cadherin, STK11, PTEN, ATM, and CHEK2 genes, while 90% of mammary tumors are adenocarcinomas with the presence of somatic mutations in high-penetrance genes such as PIK3CA, AKT1, SF3B1, GATA3, MLL3, CDH1, MAP3K1, NCOR1, MAP2K4, and MACF1 [5, 8–10]. All breast cancer's driver genes are fully detailed in the Cancer Genome Interpreter and the Pan-Cancer Atlas [11, 12]. Regarding the AKT subfamily, it is made up of three isoforms in mammals: AKT1, AKT2, and AKT3. AKT1 is located in the 14q32.33 position and has 14 exons that include a reading frame of 1443 base pairs (bp). AKT1 encodes the kinase B alpha (PKB*α*) protein of 480 amino acids and 55686 Daltons. PKB*α* contains an N-terminal pleckstrin homology (PH) domain, a short *α*-helical linker, a kinase domain (KD), and a regulatory motif [13, 14]. Various cytokines, hormones, and growth factors active AKT1 by binding their cognate receptor tyrosine kinase (RTK), GPCR, or cytokine receptor and triggering activation of the PI3K kinase, which generates PIP3 [15, 16]. AKT1 binds PIP3 through its PH domain, resulting in translocation of AKT1 to the membrane. The mTOR-rictor complex (mTORC2) phosphorylates AKT1 within the carboxy terminus at S473 and PDK1 phosphorylates AKT1 within its activation loop at T308 [16, 17]. Activated AKT1 phosphorylates a large number of downstream substrates that play a crucial role in regulating cell growth, metabolism, proliferation, apoptosis, angiogenesis, and drug responses [18–21].

**Figure 1:**
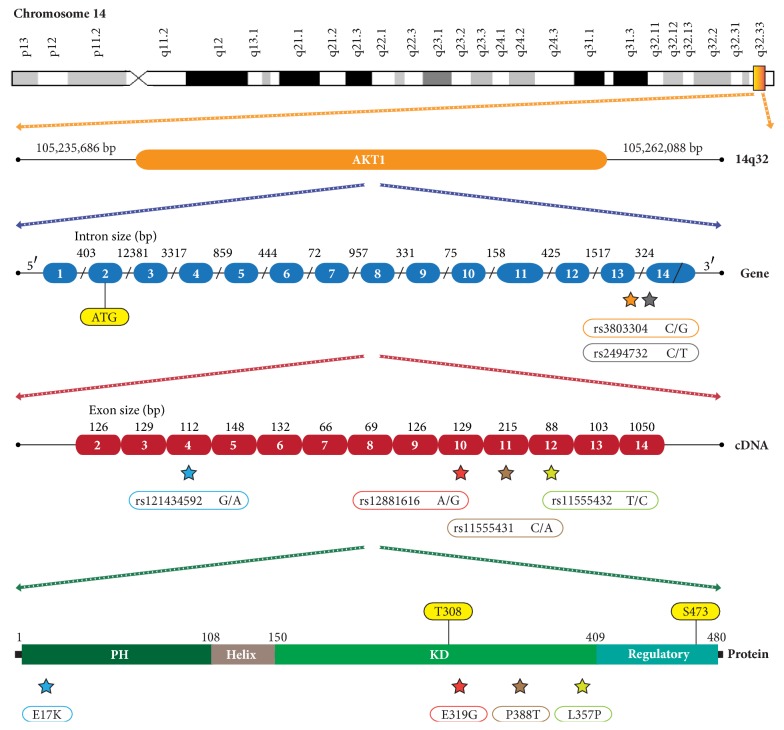
Location of the rs121434592, rs12881616, rs11555432, rs11555431, rs2494732, and rs3803304 variants in chromosome 14 in the AKT1 gene.

The E17K rs121434592, E319G rs12881616, L357P rs11555432, P388T rs11555431, rs2494732, and rs3803304 single nucleotide polymorphisms (SNPs) have been identified in the AKT1 kinase gene [22]. The E17K variant generates a conformational change in the PH domain of the AKT1 protein, making it possible to join the protein and the cell membrane, causing the process of intracellular phosphorylation [23]. The E319G, L357P, and P388T variants are located in the kinase domain (KD), and the rs2494732 and rs3803304 variants are placed in intronic regions (Figure 1). Consequently, dysregulation in cell proliferation, survival, and growth drives progressive transformation of normal cells towards a malignant phenotype [18, 24–27]. Aberrant AKT signaling is the underlying defect found in several pathologies such as esophageal cancer [28], head and neck cancer [29], and non-small cell lung cancer [30].

The objective was to determine the risk of breast cancer associated with histopathological and immunohistochemical characteristics and genetic polymorphisms in a high altitude Ecuadorian mestizo population.

## 2. Materials and Methods

### 2.1. Study Subjects

The Bioethics Committee of our institution, conducted following the Declaration of Helsinki, approved this retrospective case-control study. It comprised a total of 276 Ecuadorian mestizo women who live 2,800 meters above sea level (masl) and were included into the analysis. Concerning the individuals with BC, 91 samples from tumor tissue embedded in paraffin with luminal A, luminal B, HER2-enriched, and basal-like subtypes were obtained from the Pathology Department at Solon Espinosa Ayala Oncologic Hospital. Affected individuals were diagnosed with BC between 2008 and 2011. Each case history conferred relevant information such as age, affected breast, surgical margins, tumor stage (T1-T4), pTNM (tumor, nodule, and metastasis) classification, histopathological and molecular classification, ER status, PR status, and HER2 status. With regard to the control group, 185 peripheral blood samples from individuals of the mestizo population with no family or personal history of cancer or smoking history were selected at random from our sample collection. Thus, the matching of cases to controls presented similar age (54.0 versus 52.3 years), age at menopause (47.4 versus 46.7 years), age at menarche (14.2 versus 14.6 years), age at first live birth (26.8 versus 26.2 years), mean number of live births (2.5 versus 2.5), and breast cancer in first-degree relative (3.1 versus 2.1 percent), respectively. Furthermore, all participants included in the study signed their respective informed consent.

### 2.2. DNA Extraction and Purification

DNA extraction and purification of control and case individuals were performed using the Wizard Genomic DNA Purification Kit (Promega, Madison, WI) and the PureLink Genomic DNA Kit (Invitrogen, Carlsbad, CA), respectively. The DNA of the healthy individuals was extracted from peripheral blood samples and presented an average concentration of 135 ng/*μ*l. Meanwhile, the DNA of the affected individuals, which presented an average concentration of 84 ng/*μ*l, was extracted from ten sections (5 *μ*m) of formalin-fixed paraffin-embedded breast tumor tissue previously cut with a microtome CUT 6062 (SLEE, Mainz, Germany). Both calculations were obtained using NanoDrop 2000 (Thermo Scientific, Waltham, MA).

### 2.3. Amplification and Genotyping

Genotyping was performed using DNA sequencing analysis. A final volume of 20 *μ*l was used for each PCR reaction for AKT1 SNPs. Each reaction consisted of 16.2 *μ*l of Milli-Q water, 2 *μ*l of DNA template (10 ng/*μ*l), 0.2 *μ*M of each deoxynucleotide triphosphate (dNTP's), 3 mM of MgCl_2_, 0.05 U of Taq DNA polymerase, 2.5 *μ*l of 10X buffer (500 mM of KCl, 200 mM of Tris-HCl, pH = 8.4), and 0.4 *μ*M of forward (FW) and reverse (RV) primers detailed in Table 1.

The SNPs E17K rs121434592 (G>A) (198 bp), L357P rs11555432 (T>C) (142 bp), E319G rs12881616 (A>G) (186 bp), and P388T rs11555431 (C>A) (171 bp) presented in exonic regions and rs3803304 (C>G) (171 bp) and rs2494732 (C>T) (171 bp) presented in intronic regions were amplified through the polymerase chain reaction (PCR) technique. Supplementary Table 2 details the genetic variants analyzed in this study. The PCR program started with an initial denaturation stage lasting 5 minutes at 94°C, followed by 35 cycles of 50 seconds at 94°C, 50 seconds at different annealing temperatures (Table 1), 45 seconds at 70°C, and a final elongation for 3 minutes at 72°C. Each run was completed using a Sure Cycler 8800 thermocycler (Agilent, Santa Clara, CA). The amplified fragment was then separated by electrophoresis in 2% agarose gels stained with ethidium bromide and was observed in an ImageQuant 300 transilluminator (General Electric, Fairfield, SC).

Genotyping was performed using DNA sequencing analysis through Genetic Analyzer 3130 (Applied Biosystems, Austin, TX). The final volume of the reaction was 12 *μ*l and contained 2.8 *μ*l of Milli-Q water, 2 *μ*l of 5X buffer, 1 *μ*l of primer FW (3.2 pmol), 1 *μ*l of BigDye Terminator v3.1 sequencing standard (Applied Biosystems, Austin, TX), and 3 *μ*l of PCR product (3 to 10 ng). Once the product was amplified, it was then purified using Agencourt CleanSEQ (Beckman Coulter, Miami, FL). The amplification program consisted of 3 minutes at 96°C, followed by 30 cycles of 10 seconds at 96°C, 5 seconds at 50°C, and 4 minutes at 60°C. Finally, sequence analysis was performed using Sequencing Analysis Software 5.3.1 (Applied Biosystems, Austin, TX), and the alignment with sequences from GenBank (AKT1 NC_000014.9) was performed using Seq-Scape Software v2.6 (Applied Biosystems, Austin, TX) [7].

### 2.4. Statistical Analysis

The information from the clinical records of the patients was collected and stored in a database. Allelic and genotypic frequencies of the AKT1 SNPs were calculated; also Hardy-Weinberg equilibrium was determined by using a tool available on the Internet (http://www.oege.org/software/hwe-mr-calc.shtml) [31]. With the use of IBM SPSS Statistics 22 software (SPSS Inc., Chicago, IL), chi-square (*χ*^2^), and odds ratio (OR) (with a 95% confidence interval [CI] and 2 x 2 contingency table), tests were applied to determine the association between the risk of developing BC, the SNPs, histopathological characteristics, and immunohistochemical characteristics.* P *< 0.05 was considered statistically significant. Consequently, the false discovery rate (FDR) Benjamini/Hochberg correction was performed in order to obtain* Q* values.

## 3. Results

The distribution of baseline characteristics in all patients with BC is shown in Table 2 and is fully detailed in Supplementary Table 1. Regarding age of diagnosis, 72.7% of luminal B individuals, 58.5% of luminal A, 56.3% of basal-like, 36.4% of HER2-enriched, and 57.1% of all cases presented were 50 years old or older. The right breast was affected in 57.1% of all cases, from which 72.6% presented luminal B, 58.5% luminal A, 50% basal-like, and 45.5% HER2-enriched. Regarding T stage, 93.8% of basal-like individuals, 77% of luminal A, 72.7% of HER2-enriched, 54.5% of luminal B, and 76.9% of all cases presented T1-T2 stage. Positive LN status was presented in 51.6% of all cases. Negative ER status was presented in 35.2% of all cases, from which 100% of basal-like individuals, 100% of HER2-enriched, 9.1% of luminal B, and 7.5% of luminal A did not present this receptor (*P* ≤ 0.001;* Q* ≤ 0.05). Regarding PR status, a total of 39.6% presented a negative PR status, of which 100% was basal-like, 90.9% was HER2-enriched and luminal B, and 79.2% was luminal A (*P* ≤ 0.001;* Q* ≤ 0.05). Overall, 70.3% presented negative HER2-enriched status, of which 100% was basal-like, 79.2% was luminal A, and 54.5% was luminal B (*P* ≤ 0.001;* Q* ≤ 0.05). Finally, 67% of all cases presented positive and 33% presented negative surgical margins.

Frequency of mutations in the AKT1 gene in breast cancer subtypes is detailed in Table 2 and genotype distribution and allele frequencies of AKT1 mutations in cases and controls are detailed in Table 3. The E17K AA homozygous genotype had a frequency of 0.02 in cases and 0.00 in control and was present in 2/91 affected individuals (2.2%) and 6.25% of basal-like type (*P* > 0.05;* Q* > 0.05). The E319G GG, L357P CC, and P388T AA genotypes had a frequency of 0.00 in cases and controls and were present in 0/91 affected individuals (0%). The rs2494732 TT mutant genotype had a frequency of 0.14 in cases and 0.22 in controls and was present in 13/91 affected individuals (14.29%) and 36.4% of HER individuals (*P* > 0.05;* Q* > 0.05). The rs3803304 mutant homozygous genotype had a frequency of 0.08 in cases and 0.02 in controls and was present in 7/91 affected individuals (7.7%) and 18.2% of HER individuals (*P* > 0.05;* Q* > 0.05), whereas the rs3803304 CG heterozygous genotype was significantly different among the four subtypes (*P* ≤ 0.05;* Q* ≤ 0.05), where luminal B had the highest percentage (63.6%). Finally, the alleles of the rs2494732 and rs3803304 SNPs presented Hardy-Weinberg equilibrium.

The association between rs2494732 and rs3803304 polymorphisms and the risk of developing BC is detailed in Table 4. Regarding variant rs2494732, the CT genotype presented an odds ratio (OR) of 0.6 (95% CI = 0.3-1.0;* P* > 0.05;* Q* > 0.05); the TT genotype presented an OR of 0.4 (95% CI = 0.2-0.9;* P* ≤ 0.05;* Q* > 0.05); and the combination of CT+TT presented an OR of 0.5 (95% CI = 0.3-0.9;* P* ≤ 0.05;* Q* > 0.05). Regarding the rs3803304 intronic variant, the CG genotype presented an OR of 1.1 (95% CI = 0.6-1.9;* P* > 0.05;* Q* > 0.05); the GG genotype presented an OR of 5.2 (95% CI = 1.3-20.9;* P* ≤ 0.05;* Q* > 0.05); and the combination of CG+GG presented an OR of 1.3 (95% CI = 0.8-2.2;* P* > 0.05;* Q* > 0.05). The OR in the remaining polymorphisms was not calculated due to low and nonexisting frequency in the study population.

The association between the rs3803304 and rs2494732 polymorphisms and the histopathological and immunohistochemical characteristics is detailed in Table 5. Regarding the affected breast, tumor stage, LN status, ER status, PR status, HER2 status, and surgical margins, no statistically significant differences (*P* > 0.05;* Q* > 0.05) were found in relation to the genetic polymorphisms. Additionally, data of control individuals is fully detailed in Supplementary Table 3.

## 4. Discussion

During the last decade, breast cancer genome-wide association studies (GWAS) have identified ~80 loci with small-to-moderate effects on OR ranging from 1.05 to 1.53 [10, 32–34]. Studying SNPs is crucial to fully understand breast cancer biology, as well as for the development of novel therapeutics for cancer treatment and for providing methods for prevention and early diagnosis [35]. The most studied populations have been Asian, European, and African. However, Latin American populations have been poorly studied, making genetic characterization essential to better understand the development of BC [7, 36].

Mounting evidence exists that activation of AKT proteins is important in cancer development [14]. Hyperactivation of the AKT pathway has been detected in up to 50% of all human tumors and is closely associated with chemoresistance [37]. Therefore, AKT has been an attractive target for anticancer drug discovery [38]. In particular, genetic alterations of the AKT genes have been demonstrated in many human tumors, including breast, colorectal, and ovarian cancers [39]. Concerning BC, the activating mutations of AKT1 gene have not been widely reported, the first being a study conducted in a female Latin American mestizo population from a high altitude (2,800 masl). It is noteworthy that this retrospective research presented a limited number of cases. However, it gives us relevant information about BC risk and its association with genotype polymorphisms of the AKT1 gene.

The E17K (rs121434592) point mutation in the pleckstrin homology domain of the AKT1 gene is the major point mutation that has been reported in the literature. In 2007, Carpten et al. evaluated the complete coding regions of AKT family members for mutations in genomic DNA from clinical tumour specimens representing breast (*n* = 61), colorectal (*n* = 51), and ovarian (*n* = 50) cancers [39]. Analysis of these samples revealed a unique mutation in the PH domain of AKT1 which results in a lysine substitution for glutamic acid at amino acid 17. E17K was identified in 5 of 51 (8%) breast, 3 of 51 (6%) colorectal, and 1 of 50 (2%) ovarian cancers. Although the sample size was insufficient to document statistical significance, the lack of coincidence of these mutations indicates that the AKT1 mutation (E17K) is sufficient for pathological activation of the PI3K/AKT pathway [40]. In 2008, Stemke-Hale et al. published that E17K mutation was detected in only 6 of 418 breast cancers (1.4%), where all of them presented ER+ and PR+ [41]. In that year, Kim et al. identified the E17K mutation in 4 of 93 (4.3%) invasive ductal carcinomas [42]. En 2014, Shanti et al. used different genomic algorithms (SIFT, Polyphen 2.0, I-Mutant 2.0, and SNPs&GO) for prioritization of high-risk missense mutations in coding regions of AKT1 gene. They revealed that mutations such as E17S, E319G, L357P, and P388T were probably damaging and these mutations should be considered alongside E17K in the therapeutic development of AKT inhibitors to treat human cancer [22]. In the high altitude Ecuadorian mestizo population, the E17K mutation was found in 2 of 91 individuals (2.2%), where one of them was luminal A and the other one was basal-like (Table 2). In spite of the low percentage found in our population, it is known that the E17K (rs121434592) substitution decreases the sensitivity to an allosteric kinase inhibitor, so this mutation may have important clinical utility for AKT drug development [43].

According to the analysis of prioritization of Shanti et al., the E319G (rs12881616), L357P (rs11555432), and P388T (rs11555431) exonic variants are high-risk missense mutations of the AKT1 gene [22]. Nevertheless, these variants are 100% present in their normal homozygous state in this study due to a small sample size of the high altitude Ecuadorian mestizo population. On the other hand, the analysis of the rs2494732 and rs3803304 intronic variants was carried out. As for the rs2494732 variant, the CT genotype is found in 40.66% of the affected population with a higher percentage in the basal-like subtype (50%) and luminal A subtype (45.3%), whereas the TT genotype is found in 14.29% of the affected population with a higher percentage in the luminal A subtype (9.4%) and HER2-enriched subtype (36.4%). The OR statistical test determined that the rs2494732 variant did not present risk with the development of breast cancer in the CT heterozygous genotypes (OR = 0.6, 95% CI = 0.3-1.0;* P *> 0.05;* Q* > 0.05) or TT mutant homozygous genotypes (OR = 0.4, 95% CI = 0.2-0.9;* P* ≤ 0.05;* Q* > 0.05), just as found in the Chinese Han population with basocellular skin cancer (OR = 1.00, 95% CI = 0.5-2.2;* P* > 0.05) [41]. As a matter of fact, in this population, the T mutant allele presented a higher allele frequency (0.46) in controls than in cases (0.35), and the C normal allele had a higher allele frequency in cases (0.65) than in controls (0.54). These results prove that the presence of mutations in the rs2494732 variant is related to a protective factor in the development of breast cancer and is associated with better general survival with a hazard ratio (HR) of 0.59 (95% CI = 0.40-0.86;* P* ≤ 0.05) and progression-free survival with a HR of 0.74 (95% CI = 0.53-1.03;* P* > 0.05) in a Korean population with non-small cell lung cancer [30]. Concerning the rs3803304 variant, the CG genotype is found in 33.0% of the affected population with a higher percentage in the luminal B subtype (63.6%) and HER subtype (45.5%), whereas the GG genotype is found in 7.7% of the affected population with higher percentage in the HER2-enriched subtype (18.2%) and luminal A subtype (7.5%). The OR statistical test determined that the rs3803304 variant presented a possible risk with the development of breast cancer in individuals with the GG mutant homozygous genotype (OR = 5.2, 95% CI = 1.3-20.9;* P* ≤ 0.05;* Q* > 0.05). In this mestizo population, the C normal allele presented a higher allele frequency in controls (0.82) than in cases (0.76), and the G mutant allele had a higher allele frequency in cases (0.24) than in controls (0.18). There is a statistically significant difference between the rs3803304 CG heterozygous genotype and the four subtypes (*P* ≤ 0.05;* Q* ≤ 0.05), where the luminal B subtype was present in 63.6%. Nevertheless, it has been observed that US individuals with esophageal cancer and the rs3803304 CG genotype presented a better response to treatments with chemoradiotherapy (OR = 0.5, 95% CI = 0.25-0.99;* P* ≤ 0.05) [28]. The results suggest that the rs3803304 variant is capable of regulating DNA transcription mechanisms and, therefore, it is capable of causing risk in the development of breast cancer in the Ecuadorian mestizo population living at high altitudes.

Regarding the rs3803304 variant that presented risk associated with the development of breast cancer, the CG+GG combined genotypes were present in 11.4% of tumor stage T3-T4, in 20.9% of negative LN status, in 12.1% of negative ER status, in 14.3% of negative PR status, and in 24.2% of negative HER2 status. However, there was no statistically significant association between these combined genotypes and the histopathological characteristics (*P* > 0.05;* Q* ≤ 0.05).

According to our results, the noncoding region rs3803304 variant may act as a predictive biomarker in the risk of developing breast cancer in the high altitude Ecuadorian mestizo population. Nevertheless, sample size should be increased in future analysis to enrich statistical tests. In conclusion, this study as well as our previous genetic studies on MTHFR in breast and prostate cancer [7, 44, 45], EGFR in lung cancer [46], and GPX-1 in bladder cancer [47] is an important contribution in order to integrate pharmacogenetics in clinical practice in Ecuador and Latin America [48, 49].

## Figures and Tables

**Table 1:** Primer sequences, annealing temperatures, and genetic variations.

Polymorphisms	Primer sequences	Annealing temperatures	Location	Fragment (bp)
rs121434592 E17K	FW 5'- GGC CAA GGG GAT ACT TAC GC -3'	60	Exon 4	198
	RV 5'- AGG GTC TGA CGG GTA GAG TG -3'			

rs11555432 L357P	FW 5'- CCT TCT TGA GCA GCC CTG AA -3'	56.5	Exon 12	142
	RV 5'- TAC GAG ATG ATG TGC GGT CG -3'			

rs12881616 E319G	FW 5'- CAG AGA GGA CAC AGC ATT GCG -3'	60.5	Exon 10	186
	RV 5'- ACA AGG ACG GGC ACA TTA AGA -3'			

rs11555431 P388T	FW 5'- CAA GGA CAT CAA GCT TTG GCT -3'	61	Exon 11	171
	RV 5'- AAG TCC TTG CTT TCA GGG CT -3'			

rs3803304	FW 5'- CAA GGA CAT CAA GCT TTG GCT -3'	61	Intron 13	171
	RV 5'- AAG TCC TTG CTT TCA GGG CT -3'			

rs2494732	FW 5'- CAA GGA CAT CAA GCT TTG GCT -3'	61	Intron 13	171
	RV 5'- AAG TCC TTG CTT TCA GGG CT -3'			

bp, base pairs.

**Table 2:** Distribution (*n*, %) of baseline characteristics (at diagnosis) and frequency of AKT1 mutations in patients with all breast cancer subtypes.

	Luminal A		Luminal B		HER2-enriched		Basal-like		All		*P* value	FDR *Q* value
	*n*	%	*n*	%	*n*	%	*n*	%	*n*	%		
Age at diagnosis												

<35	1	1.9	0	0.0	1	9.1	0	0.0	2	2.2	Reference	

35-49	21	39.6	3	27.3	6	54.5	7	43.75	37	40.7	*P* > 0.05	*Q* > 0.05

≥50	31	58.5	8	72.7	4	36.4	9	56.25	52	57.1	*P* > 0.05	*Q* > 0.05

Affected breast												

Right	31	58.5	8	72.7	5	45.5	8	50.0	52	57.1	*P* > 0.05	*Q* > 0.05
Left	22	41.5	3	27.3	6	54.5	8	50.0	39	42.9		

T stage												

T1-T2	41	77.36	6	54.5	8	72.7	15	93.75	70	76.92	Reference	

T3-T4	9	16.98	5	45.5	3	27.3	1	6.25	18	19.78	*P* > 0.05	*Q* > 0.05

T0, X	3	5.66	0	0.0	0	0.0	0	0.00	3	3.30	*P* > 0.05	*Q* > 0.05

LN status												

+	28	52.8	3	27.3	9	81.8	7	43.75	47	51.6	*P* > 0.05	*Q* > 0.05
-	25	47.2	8	72.7	2	18.2	9	56.25	44	48.4		

ER status												

+	49	92.5	10	90.9	0	0.0	0	0.0	59	64.8	*P* ≤ 0.001	*Q* ≤ 0.05
-	4	7.5	1	9.1	11	100	16	100	32	35.2		

PR status												

+	53	100	1	9.1	1	9.1	0	0.0	55	60.4	*P* ≤ 0.001	*Q* ≤ 0.05
-	0	0.0	10	90.9	10	90.9	16	100	36	39.6		

HER2 status												

+	11	20.8	5	45.5	11	100	0	0.0	27	29.7	*P* ≤ 0.001	*Q* ≤ 0.05
-	42	79.2	6	54.5	0	0.0	16	100	64	70.3		

Surgical margins												

+	33	62.3	8	72.7	7	63.6	13	81.25	61	67.0	*P* > 0.05	*Q* > 0.05
-	20	37.7	3	27.3	4	36.4	3	18.75	30	33.0		

rs121434592 E17K												

GG	52	98.1	11	100	11	100	15	93.75	89	97.8	Reference	

GA	0	0.0	0	0.0	0	0.0	0	0.0	0	0.0	-	-

AA	1	1.9	0	0.0	0	0.0	1	6.25	2	2.2	*P* > 0.05	*Q* > 0.05

rs12881616 E319G												

AA	53	100	11	100	11	100	16	100	91	100	Reference	

AG	0	0.0	0	0.0	0	0.0	0.0	0.0	0	0.0	-	-

GG	0	0.0	0	0.0	0	0.0	0.0	0.0	0	0.0	-	-
rs11555432 L357P												

TT	53	100	11	100	11	100	16	100	91	100	Reference	

TC	0	0.0	0	0.0	0	0.0	0.0	0.0	0	0.0	-	-

CC	0	0.0	0	0.0	0	0.0	0.0	0.0	0	0.0	-	-

rs11555431 P388T												

CC	53	100	11	100	11	100	16	100	91	100	Reference	

CA	0	0.0	0	0.0	0	0.0	0.0	0.0	0	0.0	-	-

AA	0	0.0	0	0.0	0	0.0	0.0	0.0	0	0.0	-	-

rs2494732												

CC	24	45.3	5	45.45	6	54.5	6	37.5	41	45.05	Reference	

CT	24	45.3	4	36.36	1	9.1	8	50.0	37	40.66	*P* > 0.05	*Q* > 0.05

TT	5	9.4	2	18.18	4	36.4	2	12.5	13	14.29	*P* > 0.05	*Q* > 0.05

rs3803304												

CC	31	58.5	4	36.4	4	36.4	15	93.8	54	59.3	Reference	

CG	18	34.0	7	63.6	5	45.5	0	0.0	30	33.0	*P* ≤ 0.05	*Q* ≤ 0.05

GG	4	7.5	0	0.0	2	18.2	1	6.3	7	7.7	*P* > 0.05	*Q* > 0.05

LN, lymph node; ER, estrogen receptor; PR, progesterone receptor; HER, human epidermal growth factor receptor; FDR, false discovery rate.

**Table 3:** Genotype distribution and allele frequency of AKT1 mutations in cases and controls.

Mutations	Genotypes	Genotypic frequency			Allele frequency		
		Cases	Controls	All	Cases	Controls	All
rs121434592 E17K	GG	0.98	1.00	0.99	0.98	1.00	0.99
	GA	0.00	0.00	0.00			
	AA	0.02	0.00	0.01	0.02	0.00	0.01

rs12881616 E319G	AA	1.00	1.00	1.00	1.00	1.00	1.00
	AG	0.00	0.00	0.00			
	GG	0.00	0.00	0.00	0.00	0.00	0.00

rs11555432 L357P	TT	1.00	1.00	1.00	1.00	1.00	1.00
	TC	0.00	0.00	0.00			
	CC	0.00	0.00	0.00	0.00	0.00	0.00

rs11555431 P388T	CC	1.00	1.00	1.00	1.00	1.00	1.00
	CA	0.00	0.00	0.00			
	AA	0.00	0.00	0.00	0.00	0.00	0.00

rs2494732	CC	0.45	0.30	0.34	0.65	0.54	0.57
	CT	0.41	0.48	0.46			
	TT	0.14	0.22	0.20	0.35	0.46	0.43

rs3803304	CC	0.59	0.65	0.63	0.76	0.82	0.80
	CG	0.33	0.33	0.33			
	GG	0.08	0.02	0.04	0.24	0.18	0.20

**Table 4:** Association between rs2494732 and rs3803304 polymorphisms and breast cancer risk among cases and controls.

Mutations	Genotypes	Cases (*n* = 91), *n* (%)	Controls (*n* = 185), *n* (%)	OR	95% CI	*P* value	FDR *Q* value
rs2494732	CC ^a^	41 (45)	56 (30)	1.0	Reference		
	CT	37 (41)	89 (48)	0.6	0.3 – 1.0	*P* > 0.05	*Q* > 0.05
	TT	13 (14)	40 (22)	0.4	0.2 – 0.9	*P* ≤ 0.05	*Q* > 0.05
	CT+TT	50 (55)	129 (70)	0.5	0.3 – 0.9	*P* ≤ 0.05	*Q* > 0.05

rs3803304	CC ^a^	54 (59)	121 (65)	1.0	Reference		
	CG	30 (33)	61 (33)	1.1	0.6 – 1.9	*P* > 0.05	*Q* > 0.05
	GG	7 (8)	3 (2)	5.2	1.3 – 20.9	*P* ≤ 0.05	*Q* > 0.05
	CG+GG	37 (41)	64 (35)	1.3	0.8 – 2.2	*P* > 0.05	*Q* > 0.05

OR, odds ratio; CI, confidence interval; FDR, false discovery rate.

^a^References.

**Table 5:** Association of genotypes with histopathological and immunohistochemical characteristics.

Variables	rs3803304		rs2494732	
	CC	CG+GG	CC	CT+TT
Affected breast				

Right	28 (30.8)	24 (26.4)	24 (26.4)	28 (30.8)

Left	26 (28.6)	13 (14.3)	17 (18.7)	22 (24.2)

OR (95% CI)	0.6 (0.2-1.4)		1.1 (0.5-3.6)	

*P* value	*P* > 0.05, *Q* > 0.05		*P* > 0.05, *Q* > 0.05	

Tumor stage ^a^				

T1-T2	43 (48.9)	27 (30.7)	32 (36.4)	38 (43.2)

T3-T4	8 (9.1)	10 (11.4)	7 (7.9)	11 (12.5)

OR (95% CI)	1.9 (0.7-5.7)		1.3 (0.5-3.8)	

*P* value	*P* > 0.05, *Q* > 0.05		*P* > 0.05, *Q* > 0.05	

LN status				

+	29 (31.9)	18 (19.8)	22 (24.1)	25 (27.5)

-	25 (27.5)	19 (20.9)	19 (20.9)	25 (27.5)

OR (95% CI)	1.2 (0.5-2.8)		1.2 (0.5-2.6)	

*P* value	*P* > 0.05, *Q* > 0.05		*P* > 0.05, *Q* > 0.05	

ER status				

+	33 (36.3)	26 (28.6)	26 (28.6)	33 (36.3)

-	21 (23.1)	11 (12.1)	15 (16.5)	17 (18.7)

OR (95% CI)	0.7 (0.3-1.6)		0.9 (0.4-2.1)	

*P* value	*P* > 0.05, *Q* > 0.05		*P* > 0.05, *Q* > 0.05	

PR status				

+	31 (34.1)	24 (26.4)	25 (27.5)	30 (33.0)

-	23 (25.3)	13 (14.3)	16 (17.6)	20 (22.0)

OR (95% CI)	0.7 (0.3-1.7)		1.0 (0.4-2.4)	

*P* value	*P* > 0.05, *Q* > 0.05		*P* > 0.05, *Q* > 0.05	

HER2 status				

+	12 (13.2)	15 (16.5)	11 (8.8)	16 (20.9)

-	42 (46.2)	22 (24.2)	30 (25.3)	34 (45.1)

OR (95% CI)	0.4 (0.2-1.0)		0.8 (0.3-1.9)	

*P* value	*P* > 0.05, *Q* > 0.05		*P* > 0.05, *Q* > 0.05	

Surgical margins				

+	33 (36.3)	28 (30.8)	25 (27.5)	36 (39.6)

-	21 (23.1)	9 (9.9)	16 (17.6)	14 (15.4)

OR (95% CI)	0.5 (0.1-1.3)		0.6 (0.3-1.5)	

*P* value	*P* > 0.05, *Q* > 0.05		*P* > 0.05, *Q* > 0.05	

LN, lymph node; PR, progesterone receptor; ER, estrogen receptor; HER, human epidermal growth factor receptor.

^a^Analyses of 88 individuals.

## Data Availability

All data generated or analyzed during this study are included in this published article and its supplementary information files. Supplementary table 1 and table 3 detail clinical data of cases and controls, respectively. Clinical data is structured by genotype information, estrogen status, progesterone status, HER2/neu status, molecular subtypes, age at diagnosis, affected breast, surgical margins, lymph node status, and tumor stage. Supplementary table 2 details information about polymorphisms, nucleotide change, and amino acid change. All these data support the conclusions of the study.

## Supplementary Materials

Supplementary Materials
** Supplementary Table** **1: **genotypes and clinical data of cases.** Supplementary Table ****2: **genetic variants.** Supplementary Table ****3: **genotypes and clinical data of controls.
